# Perceptual memory drives learning of retinotopic biases for bistable stimuli

**DOI:** 10.3389/fpsyg.2014.00060

**Published:** 2014-02-03

**Authors:** Aidan P. Murphy, David A. Leopold, Andrew E. Welchman

**Affiliations:** ^1^Binocular Vision Lab, School of Psychology, University of BirminghamBirmingham, UK; ^2^Section on Cognitive Neurophysiology and Imaging, Laboratory of Neuropsychology, National Institute of Mental HealthBethesda, MD, USA; ^3^Department of Psychology, University of CambridgeCambridge, UK

**Keywords:** bistable, ambiguous figures, perceptual stabilization, cue recruitment, associative learning

## Abstract

The visual system exploits past experience at multiple timescales to resolve perceptual ambiguity in the retinal image. For example, perception of a bistable stimulus can be biased toward one interpretation over another when preceded by a brief presentation of a disambiguated version of the stimulus (positive priming) or through intermittent presentations of the ambiguous stimulus (stabilization). Similarly, prior presentations of unambiguous stimuli can be used to explicitly “train” a long-lasting association between a percept and a retinal location (perceptual association). These phenonema have typically been regarded as independent processes, with short-term biases attributed to perceptual memory and longer-term biases described as associative learning. Here we tested for interactions between these two forms of experience-dependent perceptual bias and demonstrate that short-term processes strongly influence long-term outcomes. We first demonstrate that the establishment of long-term perceptual contingencies does not require explicit training by unambiguous stimuli, but can arise spontaneously during the periodic presentation of brief, ambiguous stimuli. Using rotating Necker cube stimuli, we observed enduring, retinotopically specific perceptual biases that were expressed from the outset and remained stable for up to 40 min, consistent with the known phenomenon of perceptual stabilization. Further, bias was undiminished after a break period of 5 min, but was readily reset by interposed periods of continuous, as opposed to periodic, ambiguous presentation. Taken together, the results demonstrate that perceptual biases can arise naturally and may principally reflect the brain's tendency to favor recent perceptual interpretation at a given retinal location. Further, they suggest that an association between retinal location and perceptual state, rather than a physical stimulus, is sufficient to generate long-term biases in perceptual organization.

## Introduction

Our perception can be shaped by past sensory experiences, recent or removed in time. In vision, phenomena such as adaptation and perceptual learning illustrate that sensory experience can affect perception over different timescales (Seitz and Watanabe, 2005; Kohn, 2007). However, sensory signals are often ambiguous, and the brain must constructively process them to achieve a coherent perceptual interpretation of the environment. Bistable stimuli provide a means of dissociating sensory stimulation from perceptual experience, as there are (at least) two valid interpretations of the same sensory input (Blake and Logothetis, 2002; Sterzer et al., 2009). Using such stimuli it has been demonstrated that past perceptual experience over a range of timescales can strongly influence subsequent perception.

At short timescales, perception of bistable stimuli can be biased either toward or away from that of a recently presented unambiguous version of the stimulus, depending on the duration of the inter-stimulus interval (Nawrot and Blake, 1993; Kanai and Verstraten, 2005; Long and Moran, 2007). Similarly, brief intermittent presentations of ambiguous stimuli cause observers to repeatedly experience the same percept on consecutive presentations (Orbach et al., 1963; Leopold et al., 2002; Maier et al., 2003; Brascamp et al., 2009). This perceptual stabilization phenomenon is attributed to a putative short-term perceptual memory trace that accumulates over seconds and can last for tens of minutes (Brascamp et al., 2008; Pastukhov and Braun, 2008; Pearson and Brascamp, 2008; De Jong et al., 2012b). Over extended periods of intermittent presentation, perception alternates between phases of stability for each percept, at a rate that is inversely proportional to the interval between consecutive presentations (Brascamp et al., 2009).

At longer timescales, the resolution of bistable stimuli can be biased in a context-contingent manner through training. In a recently developed paradigm referred to as “cue recruitment,” context-dependent biases that last 24 h and are resistant to counter-training can be elicited by initial exposure to a mixture of ambiguous and unambiguous versions of the stimulus (Haijiang et al., 2006; Harrison and Backus, 2010a,b; Van Dam and Ernst, 2010; Harrison et al., 2011). Such learning has previously been described as a form of Pavlovian conditioning (Haijiang et al., 2006). In contrast to conventional notions of associative learning however, the resolution of perceptual ambiguity appears to be more important than unambiguous sensory stimulation for learning to occur (Harrison and Backus, 2010b; Van Dam and Ernst, 2010). Learning therefore appears to be driven by past perceptual experiences—internally generated interpretations of ambiguous sensory input. Here we refer to this type of long-term bias as perceptual association.

Experience-dependent changes in perception occurring at different timescales have typically been studied separately and treated as independent phenomena. Consequently the relationship between short term perceptual memory and long term perceptual associations remains poorly understood. For example, intermittent presentation of ambiguous stimuli during the training phase of perceptual association learning has been assumed to induce stabilization, but unambiguous stimuli are also typically interleaved. It therefore remains unclear to what extent the learned association between percept and retinal-location is driven by traditional associative learning mechanisms (reliant on unambiguous information) compared to learning guided by perceptual memory. Conversely, stabilization studies to date have not investigated the long-term effects of stabilization on perceptual bias.

Here we tested the hypothesis that long-term associative learning can emerge naturally from repeated instances of short-term perceptual memory, in the absence of explicit training. First we assessed the relative contribution of these two distinct processes during an extended initial “training” period of intermittent exposure to ambiguous stimuli, that is known to induce long-term perceptual associations. In the second experiment, we examined the ability of perceptual memory to reconfigure a recently learned association. The results demonstrate an important role for perceptual memory in both initiating and updating retinal location-contingent learning of perceptual biases, thus drawing a strong link between these two apparently different forms of visual plasticity. Together, these findings suggest that certain forms of long-term perceptual learning might derive from short-term mnemonic, rather than strictly associative, mechanisms. Specifically, repeated experience of a subjective perceptual state, as opposed to a physical stimulus, can give rise to an enduring bias in the subsequent interpretation of ambiguous patterns.

## Results

**Figure 1:**
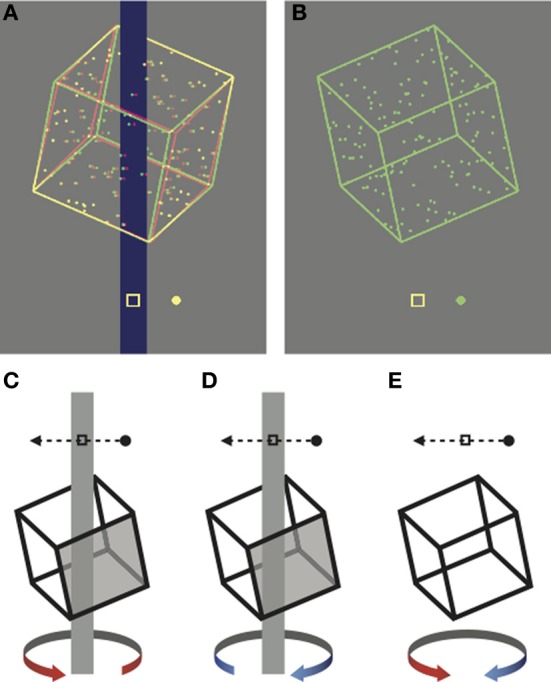
**Cropped screen shots of the stimuli, rendered here for red-green anaglyph presentation. (A)** Unambiguous stimulus disambiguated by occlusion and disparity cues. **(B)** Ambiguous stimulus presented monocularly. **(C–E)** Schematic representations of the task. Participants reported whether the front face of the cube (shaded in the unambiguous examples) moved in **(D)** the same direction as the probe dot or **(C)** the opposite direction to the probe dot. **(E)** On ambiguous trials the task was the same, but there was no correct answer.

Our experiments investigated the influence of brief visual stimulation with ambiguous and unambiguous stimuli on the establishment and maintenance of perceptual biases. In the first experiment, we investigated the influence of unambiguous stimuli in biasing subsequent perception of rotating Necker cube stimuli. The unambiguous stimuli were similar to the ambiguous versions but incorporated disparity and occlusion cues, which prompted the perception of rotation in one or the other direction (Figure 1A). We refer to the disambiguated Necker cube stimuli as *training* stimuli, as they have previously been used to train long-term biases in perception (Harrison and Backus, 2010b; Van Dam and Ernst, 2010). In an initial pilot experiment, we first replicated this long-term learning effect (Figure S1). The results confirmed that the training regimen used in the subsequent experiments was capable of inducing strong long-term learning that persisted over 24 h and was resistant to counter conditioning.

### Effects of training stimuli on perceptual bias

In the first experiment, participants viewed a succession of briefly presented ambiguously rotating Necker cubes over a block lasting approximately 20 min. Each block consisted of 400 trials, with each trial initiated by the participant with a key press. This block length was nearly four times longer than those typically used in “cue recruitment” paradigms (Figure S1; Harrison and Backus, 2010b; Van Dam and Ernst, 2010) and was selected to allow us to look for periodic perceptual dominance phases associated with perceptual stabilization (Leopold et al., 2002; Brascamp et al., 2009). On each trial the stimulus was presented either above or below the fixation point for 1.5 s. Following each presentation, the participants indicated the perceived rotation direction relative to a probe dot with a button press (see Figure 1 and Methods).

Participants were assigned to one of two conditions. In a *trained* condition (*n* = 12), the first two trials at each retinal location were unambiguous (training) stimuli. The training stimuli always rotated in opposite directions at the two locations, and were counter balanced across participants. These training trials were then followed by 396 ambiguous trials. Previous studies have suggested that initial association of unambiguous rotation direction with a retinal position leads to long-lasting, position-specific biases in perception (Haijiang et al., 2006; Harrison and Backus, 2010b; Van Dam and Ernst, 2010). To test the importance of the initial training stimuli, we also tested participants in an *untrained* condition (*n* = 7), in which the structure of the block was identical except for the absence of any training stimuli.

**Figure 2:**
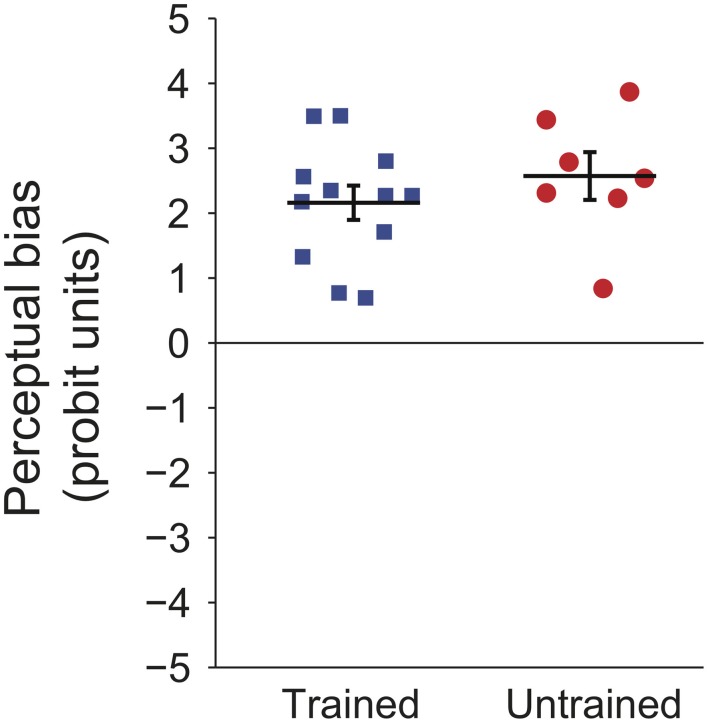
**Individual probit transformed bias summed across retinal locations for participants in the first experiment.** Black lines indicate group means and error bars show standard error.

Prominent perceptual biases emerged in both trained and untrained groups. Figure 2 shows the magnitude of this bias for each of the participants. The perceptual bias is expressed as the sum of probit transformed probabilities at each retinal location (probit units, see Methods), per earlier convention (Harrison and Backus, 2010b). For the trained group, the positive values indicate that the majority of trials were seen as moving in the trained direction at each location. For the untrained group, positive values indicate that the initial direction of bias was maintained throughout the session. The results show that the explicit training trials had no significant effect on the magnitude of the bias [*t*_(17)_ < 1, *p* = 0.37].

**Figure 3:**
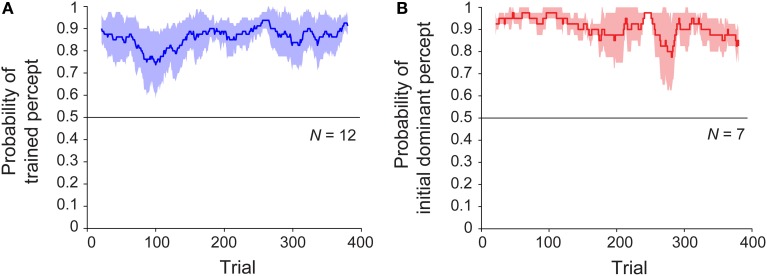
**Time course of median perceptual bias across subjects throughout the block for observers in (A) trained (*N* = 12) and (B) untrained (*N* = 7) groups.** Colored lines represent group median proportion of trials perceived in the “trained” direction for a sliding window of 40 trials, and shaded regions indicate median absolute deviation.

We next considered whether, given the initial training trials, there might be a difference in the rate of buildup or decay of the perceptual bias, as an observable effect of associative learning. To address this, we examined the time courses of sessions with and without training. Specifically, we asked whether the perceptual bias emerged gradually over the course of the session, and whether or not this change was shared across the trained and untrained conditions. The results are shown in Figure 3, which plots the probability of the trained (or initially dominant) percept as a function of trial number over the 20-min block. For the trained group, the bias in the trained direction was present from the first ambiguous trial and persisted until the end of the session (Figure 3A). Analysis using robust regression demonstrated no significant increase or decrease in this variable over time [trained group: *t*_(11)_ = 1.51, *p* = 0.16; untrained group: *t*_(6)_ < 1, *p* = 0.40]. Despite the absence of any disambiguated trials, the results for the untrained sessions were similar (Figure 3B). Note that in this case, the probability is computed relative to the initially dominant percept rather the direction of a particular training trial. For a subset of participants (*n* = 8) who were tested on a second block of 400 ambiguous trials immediately after completion of the first block, bias remained similarly stable throughout sessions lasting approximately 40 min (Figure S2).

**Figure 4:**
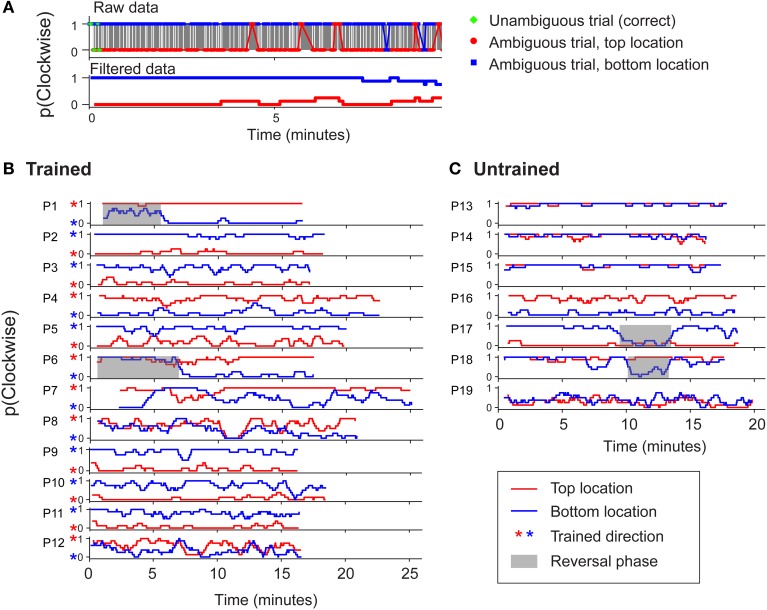
**(A)** An example to illustrate how raw perceptual report data from individual observers was boxcar filtered (with a window size of 8 trials) to enhance clarity in **(B)** and **(C)**. **(B)** Perceptual report data from observers in the trained group in experiment 1 for two consecutive blocks of 400 trials each. Red asterisks indicate the percept specified by initial disambiguated training trials at the top location. **(C)** Data from observers in the untrained group. Shaded regions indicate phases of stabilized perceptual reversal (>3 min). Such periodic alternations are characteristic of perceptual stabilization (Brascamp et al., 2009).

It has been suggested that group averaged data may be a poor measure of rate of learning (Gallistel et al., 2004). However, in agreement with our group averaged data, stable biases could be observed in the data of individual participants from both groups (Figure 4). For each participant, a boxcar-filtered time course of perceived direction is shown separately for the two retinotopic positions (red and blue traces). Figure 4B demonstrates that for the majority of participants in the trained group, training was effective for the upper and lower positions (red and blue stars, respectively). Thus two unambiguous trials at each location were generally sufficient to induce opposite-direction biases above and below the fixation point. For the untrained group (Figure 4C), biases were established early in the block and were most often in the *same* direction in the different positions. While established biases remained stable for most participants, we observed occasional phases of reversed dominance (gray highlights), which are a hallmark of perceptual stabilization (Brascamp et al., 2009). The occurrence of such reversals for several individuals contributed to the increased variability seen at certain time points in the group averaged data (Figure 3). Note that the observers in the trained group who experienced such reversals began the session reporting the untrained percept, and their subsequent reversal to the trained percept is unlikely to reflect a delayed effect of training.

These results reveal that while explicit training can determine the direction of perceptual biases, similar biases emerge in the absence of training. These spontaneous biases are of similar magnitude and stability to the trained biases and their direction can be the same or opposite at different retinal positions. The results are consistent with the suggestion that learning under the “cue recruitment” paradigm results primarily from associations formed between *percepts* and retinal locations, rather than between the physical stimuli and retinal locations, which might be predicted based on conventional theories of associative or Pavlovian learning (Harrison and Backus, 2010b; Van Dam and Ernst, 2010). From this we conclude that the formation of such long-term perceptual associations ultimately depends on perceptual memory holding the percept constant over many presentations of the physically ambiguous stimulus.

### Effects of spontaneous alternation on perceptual bias

In the experiments described above, we used a presentation paradigm that is known from previous studies as well as our own pilot experiment, to induce long-term retinal location-contingent biases (Harrison and Backus, 2010b; Van Dam and Ernst, 2010). The results suggested that perceptual stabilization (achieved through brief intermittent stimulus presentations) plays an important role in establishing such perceptual associations. Given these results, we hypothesized that perceptual alternations, which arise spontaneously during an extended period of continuous stimulus presentation, might therefore have a deleterious effect on learned biases. We thus tested whether periods of spontaneous alternation would affect recently acquired perceptual biases, and further whether they would do so in a retinotopically specific manner.

**Figure 5:**
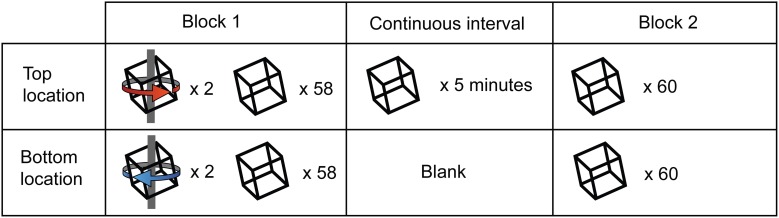
**Illustration of experiment 2 design.** All sessions began with a block of 120 trials, of which 4 out of the first 8 (2 at each retinal location) were unambiguous. Observers were then presented with an ambiguous stimulus at either the top or bottom retinal location (counterbalanced across subjects) continuously for 5 min, during which they reported perceptual alternations in the perceived direction of stimulus rotation. At the end of the continuous interval, observers completed a final block consisting of 120 ambiguous trials.

We modified the original paradigm by adding an extended period of ambiguous stimulation. Specifically we interposed 5 min of continuous rotating Necker cube presentation at one of the two retinal locations into a training block (Figure 5). During this period of continuous presentation, the opposite retinal location remained blank. A blank interval of this duration has previously been considered sufficient to demonstrate “long term” learning effects (Van Dam and Ernst, 2010). Participants reported their perceptual state for both the continuous and intermittent portions of these extended blocks. In each testing session, participants were initially biased in opposing directions for the two retinal locations, as described above. Initial analysis was restricted to those participants for whom training was effective at both locations during the first block (*n* = 13).

**Figure 6:**
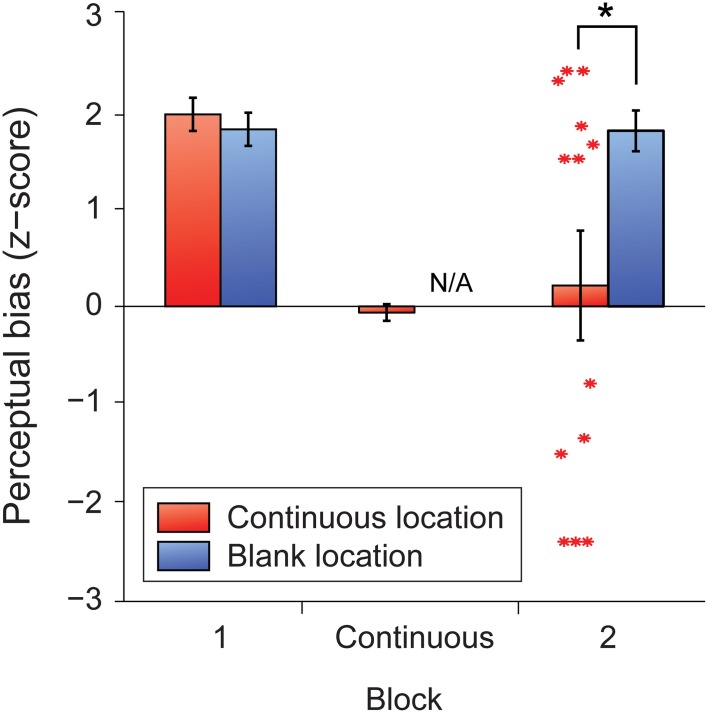
**Mean *z*-scores indicating perceptual bias in the trained direction for each retinal location for the full learners group (*N* = 13).** Error bars represent s.e.m. Red asterisks represent *z*-scores for individual participants in block 2 at the retinal location where the stimulus had previously been presented continuously. The black asterisk indicates a significant difference between means (*p* < 0.05).

We found that the five-minute period of continuous perceptual alternation effectively abolished the trained perceptual biases at the continuously stimulated location, but not at the blank location (Figure 6). Subsequently, participants expressed significant biases that were nearly equally divided between the two different directions at the continuously stimulated location (Figure 6, red asterisks). By contrast, the unstimulated retinotopic location maintained its previous bias and appeared entirely unaffected by the spontaneous alternations at the opposite location.

What factors determine the perceived direction of rotation following periods of continuous ambiguous stimulation? In the previous experiment we observed strong, stable perceptual biases from the start of the session, irrespective of whether explicit training was provided. This suggests that the perceptual interpretation of early ambiguous trials at each location determines which percept an observer becomes biased toward, since the dominant percept at the start of a session tends to become stabilized throughout. We reasoned that perhaps the last direction of rotation perceived during continuous viewing might be important. For example, the final percept experienced by an observer before the stimulus was turned off, might become stabilized during the next intermittent sequence of ambiguous trials. If this manipulation influences subsequent perceptual bias, then this would provide further evidence for the role of short-term perceptual history in long-term bias acquisition, since no disambiguating cues of any sort were applied during this phase of the experiment.

To test the dependence of the bias in the final block on the spontaneous alternation process, we related the bias direction to the ultimate perceptual state reported by all participants during the continuous presentation period. This analysis revealed that the bias in a significant majority of participants (19 of 24) matched the final perceptual state (*p* < 0.05). To test whether more distant aspects of perceptual history influenced subsequent bias, we coded final percept duration as a vector, with both magnitude (seconds) and direction relative to training (same or opposite to trained bias). This measure correlated significantly with block 2 bias (*R*^2^ = 0.17, *p* < 0.05). In contrast, we found no correlation between block 2 bias and a variety of other measures of longer-term perceptual history during the continuous presentation period (including overall bias, total perceptual alternations, frequency of perceptual alternations and bias during the final 30 s).

**Figure 7:**
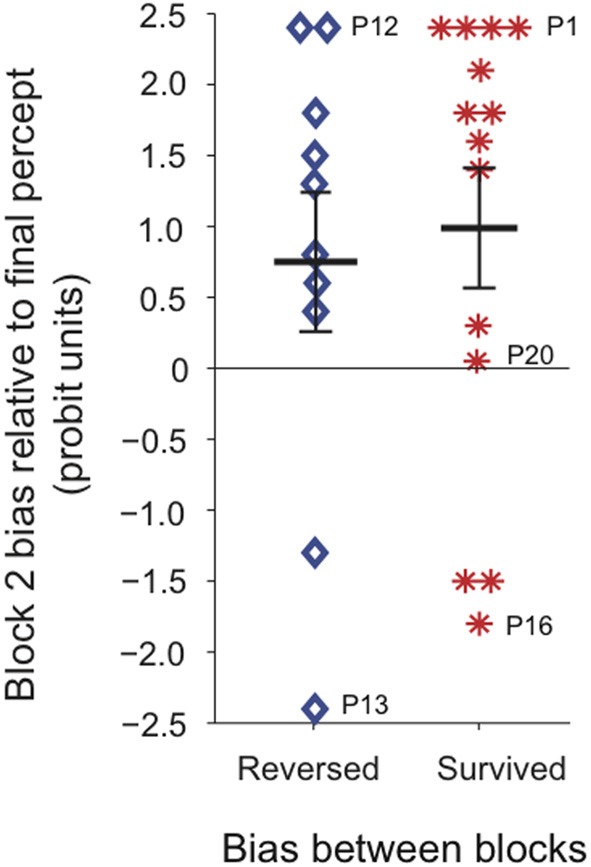
**Perceptual bias at the continuous location during block 2, for all participants tested (*N* = 24).** Participants were divided into two groups depending on whether the percept that dominated at the continuous location during block 2 was the same as (red asterisks), or opposite to (blue diamonds), the percept that had dominated at that location during block 1. Labeled data points indicate the corresponding individual observers' data. Time courses for all individual observers are shown in Figure S3. For the majority of observers the last percept experienced during continuous viewing correctly predicted subsequent perceptual bias at the continuous location.

To test whether the tendency for block 2 bias to follow the last percept experienced during continuous viewing was related to the direction of the “trained” perceptual bias prior to continuous stimulation, we divided participants into two groups depending on whether the percept that dominated at the continuous location during block 2 was the same as, or opposite to, the percept that had dominated during block 1, regardless of training (Figure 7). We found that for both groups the last continuous percept was the principal predictor of the block 2 bias direction, and there was no significant effect of prior bias direction on the magnitude of subsequent bias.

## Discussion

Perceptual resolution of ambiguous sensory input can be influenced by both recent and distant past experiences. Perceptual stabilization (Orbach et al., 1963; Leopold et al., 2002; Brascamp et al., 2008, 2009; Knapen et al., 2009) and “cue recruitment” (Haijiang et al., 2006; Harrison and Backus, 2010a,b; Van Dam and Ernst, 2010; Harrison et al., 2011) are two such examples, putatively driven by short-term perceptual memory and associative learning processes respectively. Here we tested the hypothesis that perceptual memory, rather than strictly stimulus-based associative learning, is primarily responsible for driving the learning of retinal location-dependent perceptual biases.

We assessed the stability of perceptual bias over sessions lasting up to 40 min and observed no reliable increase in bias that might reflect a gradual learning component. Instead, biases were expressed from the outset and remained relatively stable throughout, with occasional phase reversals of perceptual dominance in individual participants. This phenomenon is characteristic of perceptual stabilization (Brascamp et al., 2009), and cannot be explained by associative learning. Further, we compared perceptual bias between a group who received unambiguous training trials, and a group who received no training. We found no effect of training on the strength or stability of perceptual bias, demonstrating that such biases can emerge exclusively as a result of perceptual stabilization.

The absence of gradual changes in bias might reflect a ceiling effect, since observers in both trained and untrained groups began each session with strong, immediate biases. This result does not therefore rule out the involvement of gradual learning during training. Our results and those of previous studies demonstrate that the training paradigm used here reliably results in the learning of long-term perceptual associations, regardless of whether participants are tested 5 min (Figure 6; Van Dam and Ernst, 2010) or 24 h (Figure S1; Harrison and Backus, 2010b) after training. However, our results suggest that perception is dominated by short-term processes during training, rendering any gradual learning effects unobservable.

For observers in the untrained group, perception of the first stimulus at each location necessarily reflected “pre-existing bias.” In contrast, for the trained group, the trained percept was likely to have conflicted with observers' pre-existing biases in approximately half of all instances. (This conflict perhaps explains the occurrence of “partial learners,” for whom training failed at one of the two locations). The finding that trained and untrained observers displayed similar levels of perceptual bias therefore suggests that the relative contribution of pre-existing bias throughout the session was negligible compared to the strong effect of stabilization.

Trained biases were shown to persist following a 5 min blank interval, which has previously been considered sufficient for demonstration of “long term” learning, as opposed to stabilization (Van Dam and Ernst, 2010). Other studies have considered even shorter inter-stimulus intervals to be sufficient to diminish the contribution of stabilization (Carter and Cavanagh, 2007). Although the exact rate of decay of the short-term perceptual memory trace, which promotes stabilization, is not known, existing models predict decay to baseline within this time frame (Noest et al., 2007; Brascamp et al., 2009), and are supported by physiological evidence for multi-timescale neural adaptation (Fairhall et al., 2001). Thus the processes of short-term perceptual memory and long-term perceptual association are behaviorally distinguishable by their temporal properties. As such, the persistence of trained biases following an extended blank interval must primarily reflect long-term learning, since any contribution of residual perceptual memory will be significantly diminished over this time frame.

Following a period of continuous viewing, during which perception spontaneously alternated, observers were equally likely to exhibit a perceptual bias in the same or opposite direction to that specified by previous “training.” One way in which continuous viewing might produce this result is by causing observers to experience both percepts in approximately equal measure through frequent perceptual alternations. This period of unbiased perceptual history could serve to “reset” long-term perceptual bias, resulting in an equal likelihood of either percept dominating subsequent intermittent viewing. Another factor that could explain this result is that subsequent perception is driven by short-term factors, such as the most recent percept. We tested the relative contributions of these two factors to the observed result by comparing measures of both short term and long term perceptual history during continuous viewing with subsequent perceptual bias. We found that the last dominant percept during continuous presentation predicted subsequent perceptual bias, including its direction and to some extent its duration. This result is consistent with several previous findings regarding the probability of a previously stabilized percept regaining dominance following a short period of continuous presentation. That survival probability appears to tend toward chance as a function of the number of spontaneous alternations, and as a function of the duration of dominance of the opposing percept (Brascamp et al., 2008).

Previous findings further suggest that perceptual history preceding the last dominant percept of a presentation period influences perception at the next presentation (Brascamp et al., 2008; Pastukhov and Braun, 2008). However, we found no correlation between block 2 bias and a variety of measures of longer-term perceptual history during the continuous presentation period (including total alternations, total bias, and bias during the final 30 s). This discrepancy is likely related to the longer duration of continuous presentation used here, which resulted in a greater number of perceptual alternations. Thus, by reducing the survival probability of the previously dominant percept toward chance (“resetting”), continuous presentation increases the influence of the final perceptual state on subsequent perception. Our results therefore suggest that the effect of perceptual alternations on a recently learned perceptual association is similar to its effects on perceptual memory. Ultimately, the brain favors repeated selection of the most recently experienced percept when resolving perceptual ambiguity following a period of unstable perceptual history. This emphasizes the importance of short-term priming-like effects in determining perception, even when these conflict with recent learning.

It is important to note that the transfer of perceptual biases was asymmetrical, with those established during continuous viewing affecting perception during intermittent presentation, but not vice-versa. This result agrees with previous findings, which suggest that distinct neural mechanism underlie stochastic perceptual alternation (under continuous viewing conditions) and initial percept selection at stimulus onset (under intermittent viewing conditions) (Stanley et al., 2011; De Jong et al., 2012a). For example, individuals' perceptual alternation rate during continuous viewing is not correlated with their alternation rate during intermittent viewing (Brascamp et al., 2009). Further, pre-existing (i.e., untrained) long-term perceptual bias is observed during intermittent viewing but not continuous viewing of binocular rivalry stimuli (Carter and Cavanagh, 2007). Nevertheless, our results demonstrate that these two processes do interact, with the transfer from continuous to intermittent viewing being strong enough to disrupt a recently acquired bias.

### Classical conditioning vs. perceptual association of location-contingent biases

Experience-dependent changes in perception can occur at multiple timescales, yet they have traditionally been studied independently and considered as separate phenomena. The learning of long-lasting retinal location-contingent biases has previously been referred to as “cue recruitment” and described as a form of Pavlovian conditioning (Haijiang et al., 2006). These statements generate testable hypotheses: first, the term “cue recruitment” implies generalizability, and therefore suggests that a variety of typically uninformative signals (other than retinal location) can also be recruited by the visual system as a cue to visual appearance. Second, characterization of this learning as classical conditioning suggests that the association between percept and retinal location (i.e., the conditioned stimulus), relies on unambiguous training trials that elicit a single percept (i.e., an unconditioned response). However, the available evidence presents problems for both of these hypotheses, and instead suggests that the learning of long-term biases is strongly driven by perceptual memory.

Describing the acquisition of long-term perceptual bias as “classical” or “Pavlovian” conditioning would extend the definition of these terms in a fundamentally new direction. Modern learning theorists have previously made such extensions in order to incorporate principles of classical conditioning within a Cognitivist framework. Contemporary views of Pavlovian conditioning therefore emphasize that animals adjust by developing accurate knowledge of the environment (Rescorla, 2003). However, sensory estimation is always subject to uncertainty, so one might argue that even perception of an “unambiguous” stimulus involves some degree of interpretation, and therefore all types of association occur between “percepts.” It worth noting that our “ambiguous” and “unambiguous” stimuli do indeed lie on a continuum of perceptual uncertainty: the latter contain multiple depth cues (structure-from-motion, occlusion and binocular disparity), which are combined to minimize uncertainty. In contrast, bistable structure-from-motion stimuli contain only a single depth cue that constrains the object structure but leaves the depth order of front and back surfaces underconstrained, resulting in high uncertainty between two possible interpretations. The mapping between sensory stimulus and perceptual interpretation differs based on the degree of uncertainty, yielding qualitatively different behavioral outcomes: for the unambiguous stimuli the mapping is more or less deterministic, while for the bistable stimulus it is stochastic and strongly influenced by past experience. In the present experiments, both the dependent variable and the reinforcing stimuli are percepts derived from an ambiguous stimulus, departing substantially from even the revised classical paradigms. For this reason, we prefer to characterize the retinotopic-specific learning observed in the present study as a form of long-term ‘perceptual association’ rather than any form of classical conditioning.

### Retinotopic specificity of perceptual memory affects perceptual association learning

One striking similarity between experience-dependent biases at short and long timescales is their retinotopic specificity. Previous studies of short term biases have demonstrated that perceptual stabilization of ambiguous figures is tightly specific to retinal location; in particular, stabilization is typically disrupted if the retinal position of the stimulus is changed by more than 1° of visual angle (Chen and He, 2004; Knapen et al., 2009). Consequently, bistable stimuli presented intermittently at two sufficiently distant retinal locations will stabilize independently of each other and can therefore become stabilized in opposite percepts, as we observed for some individuals in the untrained group. A key difference between using retinal location and other novel signals to train perceptual biases is that, in the former case, only one percept is trained at a given retinal location, whereas in the latter case, two opposing percepts must be trained at the same location. Stabilization therefore promotes the learning of long-lasting perceptual associations only when retinal location is used as the novel signal, and instead interferes with learning when retinal location is kept constant. Accordingly, biases have indeed been reliably trained using retinal location as the novel signal, while various other signals appear to be ineffective (Haijiang et al., 2006; Harrison and Backus, 2010a; Jain et al., 2010). In the limited cases where non-retinal location signals have been shown to produce biases, these biases were much weaker (Haijiang et al., 2006; Jain and Backus, 2013) and either decayed rapidly after training ceased (Di Luca et al., 2010) or required special manipulations of the training condition (Harrison and Backus, 2012). This lack of generality beyond retintopic location cues is incompatible with an account of long-term bias based exclusively on conventional associative learning, but supports our finding that perceptual stabilization plays an important role in driving longer-term bias.

Retinal-location appears to be a unique signal because of its importance to perceptual stabilization, which in turn presumably relates to the retinotopic organization of visual cortex. Nevertheless, previous studies using non-retinal cues suggest that some amount of traditional associative learning (learned associations between a perceptually robust or “unambiguous” stimulus and a novel signal) can occur independently of stabilization in the training of long-term bias (Haijiang et al., 2006; Di Luca et al., 2010; Harrison and Backus, 2012; although see Pastukhov et al., 2013). The fact that observable trained biases were produced in these studies suggests that learned associations between novel signals and perceptually robust signals (unambiguous stimuli) can be significant, since stabilization effects would have interfered with training. One way to disentangle these components might be via manipulations that reduce stabilization, such as presentation timing (Leopold et al., 2002) or possibly physical stimulus properties (Brouwer and Van Ee, 2006). However, given the importance of retinal location to perceptual stabilization, it would be of interest to assess whether any of the non-retinal novel signals used in previous studies might have caused systematic changes in observers' eye movements, shifting the retinal position of stimuli in a cue-contingent manner.

### The nature of perceptual resolution

Presented with ambiguous sensory input, the visual system must constructively process signals to interpret the structure and content of the environment. Even in dynamic environments, changes typically occur on timescales comparable to the temporal resolution of visual processing, resulting in temporal autocorrelations in natural vision. One useful strategy would therefore be to re-select the most recent interpretation of the same scene. This can be seen experimentally as perceptual stabilization during intermittent presentation of a stimulus, which is analogous to the occlusion and reappearance of a moving, ambiguous object (e.g., a camouflaged predator) being tracked behind a cluttered foreground (e.g., a forest). A second adaptive strategy would be to select the interpretation of the scene that has proven most frequently to agree with other sources of sensory information in the history of one's experience (for a given context). For example, if you had witnessed similar looking moving objects in this forest before, and also heard a roar, then it might be advantageous to be biased toward perceiving the object as a predator. This corresponds to prior knowledge, which has been shown to be modified by recent sensory experience (Adams et al., 2004). In contrast, the learning of perceptual associations demonstrates that recent perceptual experience alone (i.e., internal resolutions of ambiguous sensory information) is also capable of modifying subsequent perception, despite the absence of any feedback on the reliability of the perceptual interpretation.

A recent model of adaptation postulates that perceptual statistics of the remote past are used to estimate the world's statistics (Chopin and Mamassian, 2012). According to this model, adaptation is predictive: the next dominant percept will be the one that brings the statistics of recent perceptual history closer to that of the remote past (Maloney et al., 2005; Denison et al., 2011; Chopin and Mamassian, 2012). Although the present study was concerned primarily with perceptual memory for ambiguous stimuli rather than adaptation to unambiguous stimuli, our results contrast with this idea. Instead, our results suggest that recent perceptual history can play a more important role than the statistics of the remote past when these two sources of information conflict. This might reflect differences in the way the brain estimates world statistics from past experience based on the reliability of the sensory information, i.e., the difference between the same percept arising from an ambiguous or unambiguous stimulus. Differences in the way the brain processes these stimuli are clearly evident from previous work indicating that long-term biases are more strongly driven by ambiguous than unambiguous stimuli (Harrison and Backus, 2010b).

## Conclusion

Here we investigated the contribution of short-term perceptual processes to the learning of retinal location-contingent biases for a bistable stimulus. We found that even in the absence of explicit training, bias emerges naturally as a product of stabilized perception at each location. Perceptual biases were immediate, retinotopically specific, and maintained throughout the testing session, irrespective of whether observers viewed disambiguated training trials or not. Further, we observed that a period of spontaneous perceptual alternations, induced by continuous presentation of the ambiguous stimulus, abolished the previously acquired bias. This manipulation had no effect on the bias at a second, spatially removed location, confirming the retinotopic specificity of the effect. Further analysis revealed that subsequent biases at the continuous presentation location were strongly determined by the final dominant percept reported during the continuous presentation. These results suggest that the learning of long-term biases in the perception of ambiguous stimuli relies heavily on short-term perceptual processes, which promote repeated selection of the most recent perceptual interpretation at a given retinal location.

## General methods

### Hardware and software

Stimuli were programmed in OpenGL using the Psychophysics Toolbox (Brainard, 1997; Pelli, 1997) for MATLAB™ (Mathworks, Natick, USA). In both experiments, stimuli were presented on a mirror stereoscope in a Wheatstone configuration using a pair of ViewSonic P225f CRT monitors (1600 × 1200, 100 Hz). Observers' head position was stabilized by means of a chin rest at a viewing distance of 50 cm. An EyeLink 1000 (SR Research Ltd., Ontario, Canada) infrared video eye-tracking system was used to track the position of both eyes, in order to ensure observers maintained fixation throughout the experiments.

### Stimuli

Virtual rotating cube stimuli were replications of those used by Harrison and Backus (2010b), with the exception that they were smaller in size in order to permit presentation on our stereoscope. Stimuli consisted of orthographic projections of a white wire-frame cube (Necker, 1832) on a mid-gray background. The center of the cubes were positioned either 8.5° above or below a central fixation marker, which was a 1.15 × 1.15° square outline. Cube edges subtended 6.5° when oriented in the frontoparallel plane and were 4′ wide. Each transparent face of the cube contained 25 randomly located white dots with a diameter of 6′. Cubes rotated about a vertical axis corresponding to the vertical meridian at an angular velocity of 45°s^−1^.

The starting orientation of the cubes was set by roll and pitch angles of ±25°, balanced across trials. For unambiguous trials (Figure 1A) these angles determined whether the cube appeared to be viewed from above or below at stimulus onset. On unambiguous trials, a vertical strip (0.5° wide × full screen height) was drawn through the axis of rotation to provide an occlusion depth cue and the cube was rendered stereoscopically with appropriate binocular disparity. On ambiguous trials (Figure 1B), stimuli were presented monocularly to the right eye in order to remove the cue conflict that would otherwise arise from a structure-from-motion stimulus being presented binocularly on a flat screen.

### Task

In all experiments, participants were instructed to fixate the central marker, which was always present. Participants initiated trials by key press, and following a brief delay (<0.5 s), a single rotating cube appeared, either above or below fixation. In the first experiment a probe dot appeared on each trial, which repeatedly moved through the central fixation marker at a speed of 9.15°s^−1^, either from left to right, or from right to left. The direction of the probe dot was randomized across trials in order to decouple perceived direction of cube rotation from the motor response. The probe dot was presented at zero disparity on unambiguous trials and monocularly on ambiguous trials. Participants reported (using the computer's keyboard) whether the probe dot was moving in the same direction or the opposite direction to the perceived front face of the cube (Figures 1C–E). In the second experiment no probe dot was presented and participants instead reported (via the keyboard) whether the front face of the cube moved to the left or right.

On each trial, the stimulus was presented for 1.5 s. This duration was selected based on the observation that participants in a pilot study—who were allowed to give their response at any time between 1.5 and 6 s after stimulus onset (following Harrison and Backus, 2010b)—responded on average 1.78 s (±0.07 s.e.m.) after stimulus onset. This corresponds to just 280 ms after the appearance of a cue indicating the start of the response period (a change of fixation marker color), which suggests that perceptual decisions were—on average—made within the first 1.5 s. Fixing presentation duration served to ensure that all observers experienced the same stimulus on duration, which is known to be an important parameter for perceptual stabilization. The minimum duration between consecutive stimulus presentations was set to 1s, although on average it was approximately 5.5 s for a given location since trial onset was self-paced and stimulus location was randomized. Unambiguous stimuli appearing above fixation always rotated in the opposite direction to those appearing below fixation, and this location-rotation contingency was counterbalanced across participants. In blocks that included unambiguous trials, these trials always occurred within the first 8 trials of the block. The first 8 trials therefore included every possible stimulus configuration: ambiguous and unambiguous, viewed from above and viewed from below, and located above or below fixation.

### Participants

In total, 55 naïve observers participated (16–42 years old, mean age = 21.4 years, 16 males). They were recruited through the School of Psychology at the University of Birmingham and received either £6/hr or research scheme credits for their participation. Participants had normal or corrected-to-normal vision. They provided written informed consent, in line with the ethics approval granted by the University of Birmingham's STEM ethics committee and the Declaration of Helsinki.

Data from participants in the trained group of experiment 1 who exhibited overall perceptual biases opposite to that expected based on training at either retinal location, were excluded from further analysis (*N* = 4). In such cases, perception may have initially been biased by a strong pre-existing bias or a negative aftereffect from the preceding unambiguous stimulus, but in either case could not be considered “trained.” For experiment 2, participants were divided into two groups based on whether training successfully influenced perceptual bias during block 1. All participants correctly identified the direction of rotation on unambiguous training trials. Eye movement data revealed that all participants tested were largely successful in maintaining fixation during stimulus presentations.

### Analysis

To analyse the time course of perceptual bias, data were boxcar filtered using a sliding window of 40 trials (20 trials at each stimulus location). For each window position, the proportion of trials for which the reported direction of rotation matched the direction specified by disambiguated training trials at the given location was calculated. We use the term ‘perceptual bias’ here to refer to the probability of experiencing one percept over the other. We make the assumption that *on average across individuals*, untrained observers begin with an equal probability of perceiving either percept.

To facilitate comparison of our results with those from previous studies, the proportion of ambiguous stimuli reported as rotating in the direction specified by disambiguated stimuli at each location were converted into z-scores using a probit transformation (inverse cumulative distribution function) (Harrison and Backus, 2010b). Saturated values (probabilities of 0 or 1) were substituted with maximum z-scores equivalent to one response in the opposite direction to training per block (±2.394 in experiment 2). The sum of the z-scores for the two locations was calculated for each subject, to give a measure of training-induced perceptual bias. All analyses were conducted using MATLAB™ (Mathworks, Natick, USA); in particular, robust regressions were implemented using the built-in function *robustfit.m*.

## Author contributions

Aidan P. Murphy and Andrew E. Welchman conceived and designed the study. Aidan P. Murphy performed experiments. Aidan P. Murphy and Andrew E. Welchman analyzed the data. Aidan P. Murphy, Andrew E. Welchman, and David A. Leopold interpreted the data. Aidan P. Murphy drafted the manuscript and prepared figures. Aidan P. Murphy, David A. Leopold and Andrew E. Welchman edited and revised the manuscript.

### Conflict of interest statement

The authors declare that the research was conducted in the absence of any commercial or financial relationships that could be construed as a potential conflict of interest.

## Supplementary material

Figure S1 **Time course of perceptual bias in the pilot experiment, which was a replication of Harrison and Backus's (2010b) “uninformative” training group. (A)** On day 1, observers (*n* = 8) completed 4 blocks of 120 trials. Each block began with 2 unambiguous trials (within the first 4 trials) at each retinal location, and observers took 2 min breaks between blocks. The time course reveals that strong biases were expressed immediately and persisted across blocks. However, the short duration of each block and the presence of unambiguous trials at the start of each block made the relative contributions of short-term (perceptual memory) and long-term (associative learning) processes difficult to assess. **(B)** On day 2, observers (*n* = 6) again completed 4 blocks of 120 trials, but now 50% of trials were unambiguous and specified the opposite direction of rotation to day 1 training at each location. Black lines indicate robust regression fits to data from all 4 blocks in each session. **(C)** Overall perceptual bias in trained direction for ambiguous trials across days for individual observers. Despite attempted “counter-conditioning” on day 2, the biases specified by day 1 training were, overall, even stronger than on day 1, although this difference did not reach significance. This result confirms that the training regimen employed in Experiments 1 and 2 was capable of eliciting robust, long-term learning effects, as previously described in “cue recruitment.”

Figure S2 **Time course of perceptual bias for a subset of participants in the “trained” group of experiment 1 (*N* = 8), who performed two “superblocks” of 400 trials each**. Biases remained stable throughout the sessions, which took participants approximately 40 min to complete.

Figure S3 **Time courses of perceptual bias for individual participants in experiment 2. (A)** Full learners—participants who expressed biases in the trained direction at both locations. **(B)** Partial learners—participants who expressed a bias in the trained direction at only one location. For clarity, raw data was boxcar filtered with a window of 8 trials for intermittent blocks or 28 s for continuous blocks. Red asterisks indicate the direction specified by unambiguous “training” trials at the top location in block 1. Diamond symbols indicate the last reported percept during the continuous block, and their color indicates whether block 2 bias matched that percept (green = yes, red = no). **(B)** Observers in the partial learners group only acquired the “trained” bias at one retinal location during block 1. Note that all participants correctly identified the direction of rotation for the unambiguous “training” trials, which were presented at the start of block 1. Despite failure to bias perception in the direction specified by training, perception readily stabilized in the opposite direction.
