# Supplementary figures and images for: Perceptual memory drives learning of retinotopic biases for bistable stimuli
Source: Front Psychol. 2014 Feb 3;5:60. doi: 10.3389/fpsyg.2014.00060 (PMC3909828; doi:10.3389/fpsyg.2014.00060)

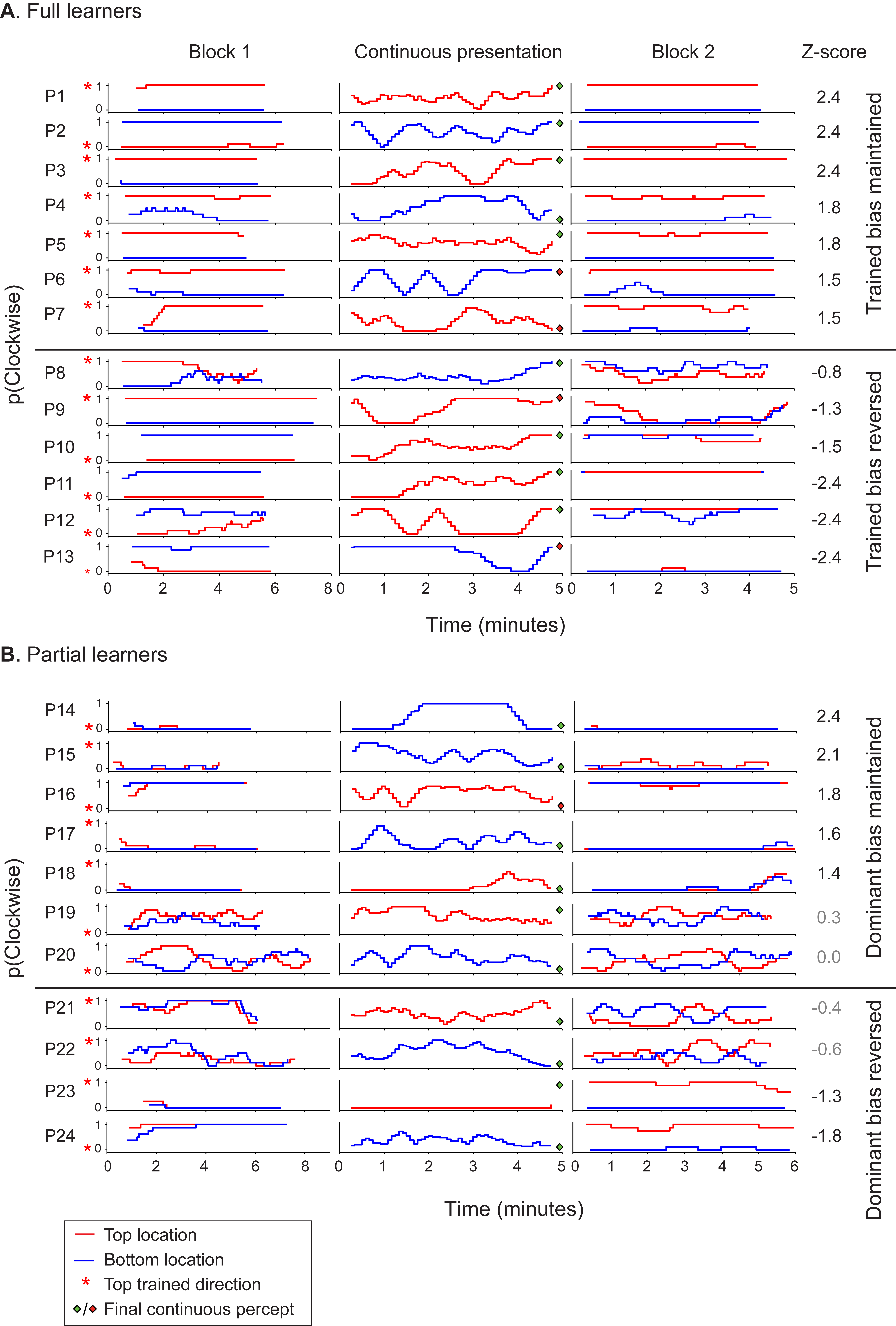

Supplement: Figure S1 — Time course of perceptual bias in the pilot experiment, which was a replication of Harrison and Backus's (2010b) “uninformative” training group. (A) On day 1, observers (n = 8) completed 4 blocks of 120 trials. Each block began with 2 unambiguous trials (within the first 4 trials) at each retinal location, and observers took 2 min breaks between blocks. The time course reveals that strong biases were expressed immediately and persisted across blocks. However, the short duration of each block and the presence of unambiguous trials at the start of each block made the relative contributions of short-term (perceptual memory) and long-term (associative learning) processes difficult to assess. (B) On day 2, observers (n = 6) again completed 4 blocks of 120 trials, but now 50% of trials were unambiguous and specified the opposite direction of rotation to day 1 training at each location. Black lines indicate robust regression fits to data from all 4 blocks in each session. (C) Overall perceptual bias in trained direction for ambiguous trials across days for individual observers. Despite attempted “counter-conditioning” on day 2, the biases specified by day 1 training were, overall, even stronger than on day 1, although this difference did not reach significance. This result confirms that the training regimen employed in Experiments 1 and 2 was capable of eliciting robust, long-term learning effects, as previously described in “cue recruitment.” [file Presentation1.ZIP › 40377_Murphy_Supplementary Figure_3.TIF]

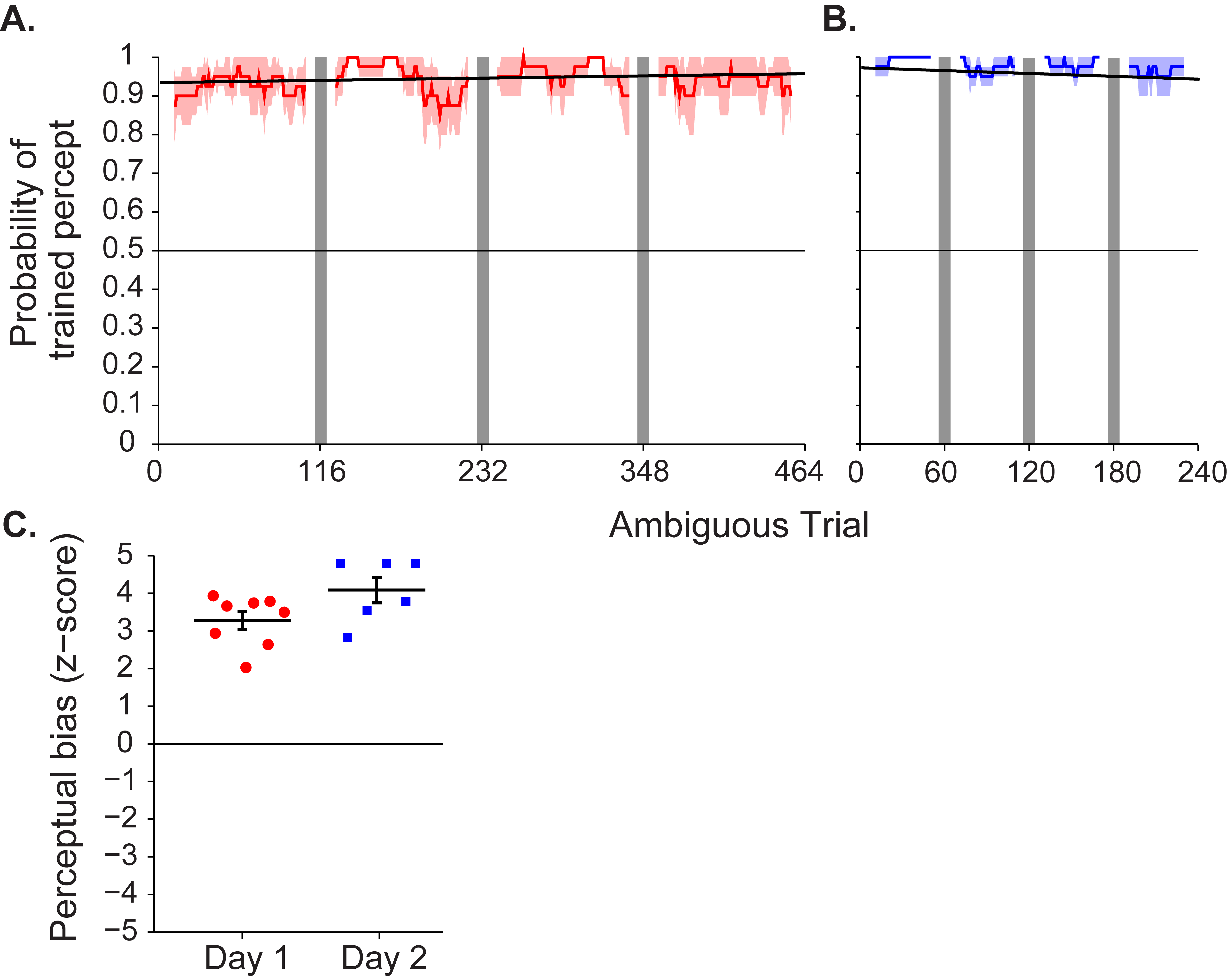

Supplement: Figure S1 — Time course of perceptual bias in the pilot experiment, which was a replication of Harrison and Backus's (2010b) “uninformative” training group. (A) On day 1, observers (n = 8) completed 4 blocks of 120 trials. Each block began with 2 unambiguous trials (within the first 4 trials) at each retinal location, and observers took 2 min breaks between blocks. The time course reveals that strong biases were expressed immediately and persisted across blocks. However, the short duration of each block and the presence of unambiguous trials at the start of each block made the relative contributions of short-term (perceptual memory) and long-term (associative learning) processes difficult to assess. (B) On day 2, observers (n = 6) again completed 4 blocks of 120 trials, but now 50% of trials were unambiguous and specified the opposite direction of rotation to day 1 training at each location. Black lines indicate robust regression fits to data from all 4 blocks in each session. (C) Overall perceptual bias in trained direction for ambiguous trials across days for individual observers. Despite attempted “counter-conditioning” on day 2, the biases specified by day 1 training were, overall, even stronger than on day 1, although this difference did not reach significance. This result confirms that the training regimen employed in Experiments 1 and 2 was capable of eliciting robust, long-term learning effects, as previously described in “cue recruitment.” [file Presentation1.ZIP › 40377_Murphy_Supplementary Figure_1.TIF]

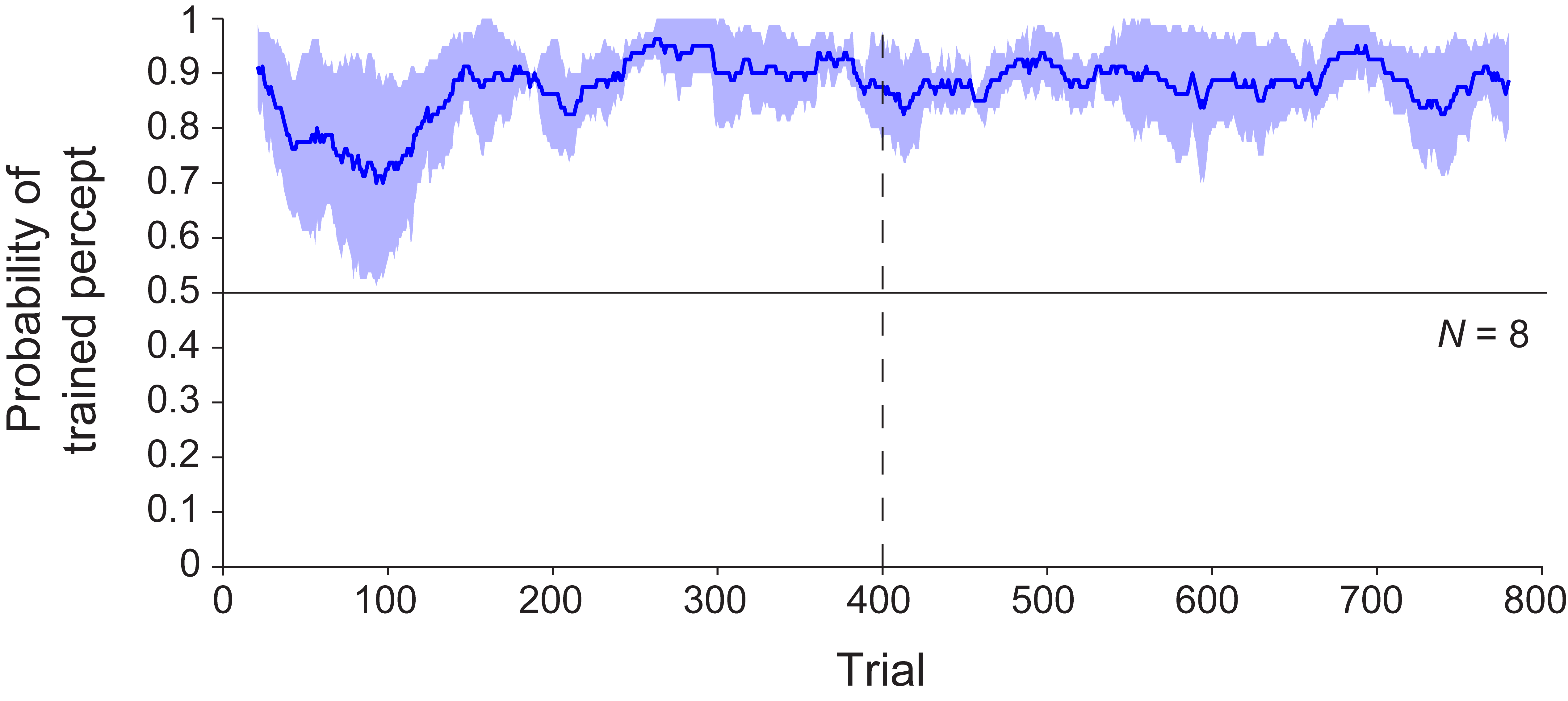

Supplement: Figure S1 — Time course of perceptual bias in the pilot experiment, which was a replication of Harrison and Backus's (2010b) “uninformative” training group. (A) On day 1, observers (n = 8) completed 4 blocks of 120 trials. Each block began with 2 unambiguous trials (within the first 4 trials) at each retinal location, and observers took 2 min breaks between blocks. The time course reveals that strong biases were expressed immediately and persisted across blocks. However, the short duration of each block and the presence of unambiguous trials at the start of each block made the relative contributions of short-term (perceptual memory) and long-term (associative learning) processes difficult to assess. (B) On day 2, observers (n = 6) again completed 4 blocks of 120 trials, but now 50% of trials were unambiguous and specified the opposite direction of rotation to day 1 training at each location. Black lines indicate robust regression fits to data from all 4 blocks in each session. (C) Overall perceptual bias in trained direction for ambiguous trials across days for individual observers. Despite attempted “counter-conditioning” on day 2, the biases specified by day 1 training were, overall, even stronger than on day 1, although this difference did not reach significance. This result confirms that the training regimen employed in Experiments 1 and 2 was capable of eliciting robust, long-term learning effects, as previously described in “cue recruitment.” [file Presentation1.ZIP › 40377_Murphy_Supplementary Figure_2.TIF]
